# Ending AIDS deaths requires improvements in clinical care for people with advanced HIV disease who are seriously ill

**DOI:** 10.1016/S2352-3018(23)00109-1

**Published:** 2023-06-07

**Authors:** Rachael M Burke, Nicholas Feasey, Ajay Rangaraj, Maria Ruano Camps, Graeme Meintjes, Wafaa M. El-Sadr, Nathan Ford

**Affiliations:** 1 Malawi Liverpool Wellcome Research Programme, Blantyre, Malawi; 2 Clinical Research Department, London School of Hygiene and Tropical Medicine, London, UK; 3 Liverpool School of Tropical Medicine, Liverpool, UK; 4 Global HIV, STIs and Hepatitis Programme, World Health Organisation, Geneva, Switzerland; 5 I-TECH, Blantyre, Mozambique; 6 Department of Medicine, Wellcome Centre for Infectious Diseases Research in Africa and Institute of Infectious Disease and Molecular Medicine, University of Cape Town, South Africa; 7 ICAP, Mailman School of Public Health, Columbia University, New York, USA; 8 Centre for Infectious Disease Epidemiology and Research, School of Public Health and Family Medicine, University of Cape Town, Cape Town, South Africa

## Abstract

Over 4 million adults are living with advanced HIV disease with approximately 650 000 fatalities from HIV reported in 2021. People with advanced HIV disease have low immunity and can present to health services in two ways: those who are well but at high risk of developing severe disease, and those who are severely ill. These two groups require specific management approaches that place different demands on the health system. The first group can generally be supported in primary care settings but require differentiated care to meet their needs. The second group are at high risk of death and need focused diagnostics and clinical care, and possibly hospitalisation. Investments in high-quality clinical management of patients with advanced HIV disease who are seriously ill at primary care or hospital level (often only for a brief period of time during their acute illness) improves the likelihood that their condition will stabilise and that they will recover. Providing high-quality and safe clinical care that is accessible to these groups of people living with HIV who are at risk of severe illness and death is a key priority for reaching the global target of zero AIDS deaths.

## Introduction

The public health approach to HIV care, first set out by the World Health Organization in 2003,^1^ has been instrumental in making antiretroviral therapy (ART) accessible to millions of people and has saved many millions of years of life.^2^ Twenty years into the public health approach, it remains important to ensure that the care available can also meet the needs of the subset of individuals who require complex or individualised clinical care, with attention paid to access and equity. An estimated 4.3 million adults are living with advanced HIV disease^3^ and 650,000 people died from HIV in 2021.^4^ All people living with HIV aged over five years with a CD4 count <200 cells/mm^3^ are considered to have advanced HIV disease, putting them at greater risk of developing severe opportunistic infections and death.^5^ Advanced HIV disease (AHD) is a challenge among people who are diagnosed late and start ART at an advanced stage of disease progression, in people who have not yet started ART, and in people who have had ART interruptions or ART failure for a range of reasons.^6^

People with AHD broadly speaking can present to health services in two ways: those who are at high risk of developing severe disease but are currently relatively well with no or few symptoms, and those who are severely ill. These two groups require different management approaches that place different demands on the health system.

### Two routes to care

The first group of people with AHD – those at high risk of developing severe disease – have few symptoms and can generally be supported in primary care settings, but require differentiated care to meet their needs. It is imperative that such people are identified early and before the development of serious symptoms, and are provided with a package of care that includes rapid ART (reinitiation), screening for tuberculosis and cryptococcal disease, prophylactic and pre-emptive treatments, and adherence support. The core package of care for this group of individuals recommended by WHO is described in the 2017 Advanced HIV Disease Guidelines.^7^ Guidance on management strategies appropriate for this group has been informed by the REALITY^8^ and REMSTART^9^ trials, both of which showed survival benefit among individuals who received a packages of care including cryptococcal antigen screening, co-trimoxazole, fluconazole, azithromycin and TB preventive treatment. However, people with AHD have been shown in several studies not to benefit from empiric TB treatment.^10–12^ It should also be noted that this group of patients may require more frequent follow up and adherence support than the general population of people without AHD on ART, but do not always require individualised care.

The AHD package of care might need to be adapted to include additional interventions addressing screening for infections of regional geographical importance, such as histoplasmosis in Latin America ^13^ or talaromycosis in SouthEast Asia.^14^

The second group – those with serious illness – need focused diagnostics and clinical care, and possibly hospitalization. People who are seriously ill can be identified by WHO danger signs: for adults these are tachypnoea, tachycardia and inability to walk unaided; for children these are lethargy, convulsions, inability to drink or breastfeed and repeated vomiting.^5^ Such individuals are seriously ill and are at extremely high risk of death. This group should also receive screening for TB and cryptococcal disease and other components of the AHD package of care as described above, but they also require healthcare-worker directed specific diagnostic testing for a broader range of opportunistic conditions, judicious use of empirical therapy (including antibiotics) and tailored supportive management (such as supplemental oxygen or intravenous fluids) whilst acutely ill.

There is relatively little epidemiological information on people with AHD who are seriously ill or admitted to hospital as this group is often excluded from large community or clinic based primary care trials or prospective cohorts. A systematic review of published hospital cohorts showed that the leading causes of death for adults and children were AIDS-related illness and severe bacterial infections.^15^ Other common causes of hospital admission include respiratory infections, neuropsychiatric presentations and renal disease for adults and malaria, haematological conditions and malnutrition for children.^15^ The same review found that fifth (20%) of adults and 14% of children living with HIV admitted to hospital died during their hospital admission.^15^ Another review found that of those who survived their hospital admission, a third died (14%) or were readmitted to hospital (19%) after discharge.^16^ In a study from Blantyre, Malawi, approximately a quarter of all adult HIV-related deaths in 2018 occurred among inpatients in the single large government hospital. Tackling advanced HIV disease in people who are seriously ill is crucial to end AIDS deaths and an area of unmet need, and the identification of interventions to reduce inpatient mortality is a research priority^17,18^ Investments in high quality clinical management of patients with AHD who are seriously ill, often only for a brief period of time during their acute illness, improves the likelihood that their condition will stabilise and that they recover, enabling them to experience the benefit of long term ART which can add decades of life.

## Clinical needs

Patients with AHD have complex medical management needs. The benefits and harms of various interventions for this population are not well studied and are made less predictable both because multiple opportunistic infections often co-exist^19^ and because of diagnostic limitations.

Diagnostic testing for people with AHD who are seriously ill includes pathogen specific tests such as near point of care molecular tests for TB on sputum and non-sputum samples, and non-disease-specific tests such as radiological tests (chest X-ray and ultrasound) and basic biochemistry and haematology tests. Supportive treatments for patients who are acutely ill and have AHD such as fluids and supplemental oxygen are often necessary, although these treatments have not been systematically studied in this patient population. There are relatively few studies assessing interventions for people who are seriously ill and have AHD, with findings from trials focusing on tuberculosis diagnosis,^20,21^ and cryptococcal meningitis^22^ and with ongoing trials in cryptococcal meningitis (ISRCTN15668391) ^23,24^ and disseminated tuberculosis (NCT04951986).^25^ Pragmatic trials of the management of acute febrile adult medical patients are needed in African settings. In order to improve outcomes for these patients, individualised input from experienced clinicians is usually needed to integrate information from symptoms, signs, laboratory and radiological testing to develop an individually tailored treatment plan. Skilled nursing practitioners in sufficient numbers are needed to monitor and care for these seriously ill patients. Such care should be holistic, including providing symptom relief and support for rehabilitation and palliative care as needed. Providing holistic care means taking into account social and psychological factors that might contribute to ill health or poor access to care and working alongside patients, their caregivers (in the case of children) and their families to focus on what is most important to them. The healthcare team should provide a welcoming and non-judgemental environment, particularly for people who are re-engaging with healthcare after a period away from ART care. Adults admitted to hospital often face catastrophic costs^26^ and poverty can be associated with higher risks of hospitalisation;^27^ if possible, health systems could consider ways to ameliorate catastrophic costs incurred by people who are seriously unwell with HIV or their caregivers or families.

The two groups of people with AHD – those at high risk and those who are seriously ill – may overlap and individuals can move between groups: people who are asymptomatic but with a very low CD4 cell count can rapidly become seriously ill, and after initial clinical management people recovering from serious illness will benefit from ongoing prophylaxis, differentiated service delivery and holistic supportive community care, perhaps including home visits. Thus, it is particularly important to identify virologic failure and the reasons for it, such as poor adherence, need to be determined and addressed. In the REALITY trial, adults and children with CD4 <100 cells/mm^3^ were recruited mostly as outpatients (i.e. started off as high risk) but over half had a serious adverse event and 13% died in first 48 weeks after initiation of ART.^8^ In another trial, nearly a quarter of adults initially recruited as high risk ambulatory outpatients starting ART with low CD4 cell count were subsequently admitted to hospital within six months.^28^ It is therefore important to develop strategies that address both groups of patients with AHD and recognise that patients transition between groups.

## Conclusions

WHO has developed a policy brief summarising recommendations for care for people living with HIV who are seriously ill, focusing on hospital-based care.^29^ This policy brief complements the existing AHD package of care, which is more focused on patients with low CD4 cell counts but have few symptoms. The WHO policy brief aims to assist national HIV programmes, hospitals, and organisations that provide care for people living with AHD to plan clinical care services for those who are seriously ill. The document summarises recommended diagnostic testing and treatments and provides considerations for organising healthcare system networks to enable access to clinical advice, allow timely and appropriate referral, and improve post-discharge care including linking from hospital level to primary care clinic or community level services.

Preventing deaths among people with AHD, whether at high risk of serious illness or who are seriously ill is a critical component to achieving the goals of ending AIDS as a public health problem by 2030. Providing high quality and safe clinical care that is accessible to these groups of people living with HIV who are at risk of severe illness and death is a key priority in order to get closer to reaching the global target of zero AIDS deaths.
